# Multi-Level Scale Attention Fusion Network for Adhesive Spots Segmentation in Microlens Packaging

**DOI:** 10.3390/mi16091043

**Published:** 2025-09-11

**Authors:** Yixiong Yan, Sijia Chen, Lian Duan, Dinghui Luo, Fan Zhang, Shunshun Zhong

**Affiliations:** 1 College of Mechanical and Vehicle Engineering, Changsha University of Science & Technology, Changsha 410114, China; 2 State Key Laboratory of Precision Manufacturing for Extreme Service Performance, Central South University, Changsha 410083, China

**Keywords:** adhesive spots segmentation, multi-level attention, multi-scale channel-guided

## Abstract

The demand for high-quality beams from high-power lasers has led to the need for high-precision inspection of adhesion points for collimating lens packages. In this paper, we propose a Multi-Level Scale Attention Fusion Network (MLSAFNet) by fusing a Multi-Level Attention Module (MLAM) and a Multi-Scale Channel-Guided Module (MSCGM) to achieve highly accurate and robust adhesive spots detection. Additionally, we built a Laser Lens Adhesive Spots (LLAS) dataset using automated lens packaging equipment and performed pixel-by-pixel standardization for the first time. Extensive experimental results show that the mean intersection over union (mIoU) of MLSAFNet reaches 91.15%, and its maximum values of localization error and area measurement error are 21.83 μm and 0.003 mm^2^, respectively, which are better than other target detection methods.

## 1. Introduction

Adhesive spot detection technology has been widely used in precision dispensing fields such as optoelectronic packaging and the automotive industry [1,2,3,4]. The quality of the adhesive spots directly affects the bonding performance of the components, which in turn has an impact on the service life of the device. In the packaging of laser diode collimating lenses, the adhesive spot securing the microlens typically has an area of only 0.1–0.4 mm^2^, making it highly dependent on manual inspection, which results in low efficiency. At the same time, the stress difference after curing can cause slight displacement of the microlens, which can significantly affect the laser beam quality if the adhesive spots areas are not uniform. Currently, the detection of small-scale optical images, such as adhesive spot segmentation, has become a crucial research direction in the field of image recognition, which shows great potential for application in automated equipment and has attracted significant attention from researchers.

In the past few years, research aimed at adhesive spot detection has been gradually applied to the field of industrial automation and achieved certain results. In the traditional model-driven method, Legrand separated the adhesion region inside the plastic cap from the background based on filter denoising and threshold segmentation methods to realize adhesive spot detection in 2002 [5]. Haniff developed a real-time and efficient gluing defect matching system in 2011 by combining vision algorithm sensors with three specially designed defect templates for the detection of adhesive spot defects in industrial electronics [6]. Zhao combined the comprehensive evaluation method based on entropy-weighted fuzzy with support vector machine (SVM) in machine learning to establish a mapping model between dispensing index and dispensing quality grade, thus realizing the online intelligent evaluation of dispensing quality [7]. Yang employed the Otsu algorithm to segment the dispensing raw image, and then applied the particle swarm optimization (PSO) algorithm to optimize the speed and accuracy of the image segmentation, which achieved an increase in the speed of dispensing image processing in 2015 [8]. However, traditional model-driven-based adhesive spot detection algorithms often exhibit poor detection accuracy when facing multi-scale adhesive spot detection in complex backgrounds, leading to their poor performance in the field of microlens packaging.

With the rapid development of data-driven neural networks, its powerful image segmentation capability makes it more efficient and accurate in segmenting complex images [9,10]. Peng accomplished the segmentation of adhesive spot contours and trajectories in pad elements using a Mask RCNN-based segmentation network in 2024, which solved the problems of robustness and dispensing accuracy in the segmentation of dispensing images [11]. Li proposed a segmentation method for adhesive spot in compact camera module images based on U-net-improved real-time segmentation network in 2023, which improves the real-time and accuracy of dispensing image segmentation [12]. Zhang used a combination of Cycle-GAN model and VGG16 model to achieve image segmentation of UV-coated glues used for lenses and mounts in the assembly process of automotive cameras in 2022, which greatly improves the accuracy of glue spot segmentation [13]. Ma designed an edge-aware defect segmentation model and trained the model using reconstructed training images. The method effectively improves the robustness of the model to distributional differences and achieves satisfactory results in the task of rail surface defect segmentation [14]. The above methods illustrate the great potential of deep learning methods in the field of adhesive spots detection; however, there is a lack of in-depth research on the micro-scale adhesive spots detection side for microlens packages.

**Figure 1:**
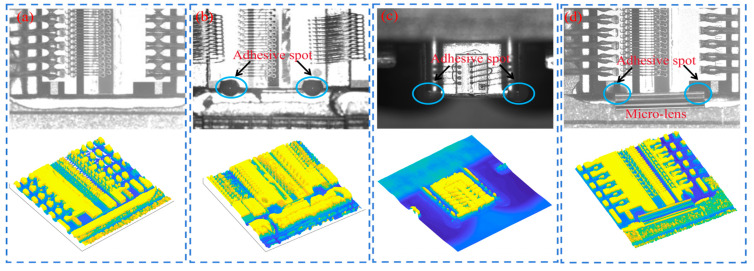
(**a**) Laser chip without adhesive spot, (**b**,**c**) laser chip with adhesive spot, (**d**) laser chip with packaged microlens.

Semiconductor lasers present divergent beams, which need to be shaped using a collimating lens at the emitting surface. Due to the small size of the laser chip, the size of the adhesive spots used to fix the microlens is limited. Figure 1 illustrates the location of the double adhesive spots on the surface of the laser chip and the fixation of the microlens.

As we can see from Figure 1, the adhesive area is heavily disturbed by the laser diode background, which makes the recognition process difficult. In the laser diode-fronted micro-optics packaging process, the area containing the adhesive spots must first be accurately identified to prevent the amount of adhesive from being too much and obscuring the functional area of the chip. Then, the location of the adhesive spot needs to be accurately detected to ensure the accuracy of the microlens gluing position. The size of the adhesive spot area directly affects the cemented position of the microlens, which in turn affects the collimation quality of the laser beam. Therefore, the accurate detection of the area and position of the adhesive spots in Figure 1 is a challenging task, and the high-precision quality detection technology of the adhesive spots is of great significance for the package of microlenses.

Inspired by the application of deep learning in image recognition, in order to accurately recognize the adhesive spots on laser chips, we first built a dataset of high-quality laser chip adhesive spots named LLAS. Then, an embedded multi-level attention network based on U-net is established to improve the information extraction ability of feature maps under different levels. Meanwhile, in order to enhance the channel information interaction capability between different feature maps, MSCGM is designed for realizing the channel-guided information interaction capability and improving the robustness of adhesive spots recognition. The main contributions of this paper include the following:

1. For the first time, we have constructed LLAS, a high-quality, pixel-by-pixel, labeled adhesive spots dataset for high-performance packages of microlenses, which strongly contributes to the field of quality inspection of micro-optical components.
2. Aiming at the characteristics of random shape, multi-scale area, and complex background of the adhesive spots, MLSAFNet is proposed to improve the feature fusion capability and detection robustness. The information interaction and feature enhancement ability under multi-level and multi-scale are enhanced by embedded MLAM and MSCGM to improve the positive detection rate of adhesive spots.
3. By comparing with the current state-of-the-art target detection algorithms, MLSAFNet shows more obvious advantages in the detection results, and realizes high-precision detection for the on-site adhesive spots images collected under complex conditions.

## 2. Methodology

### 2.1. The Whole Model Structure

**Figure 2:**
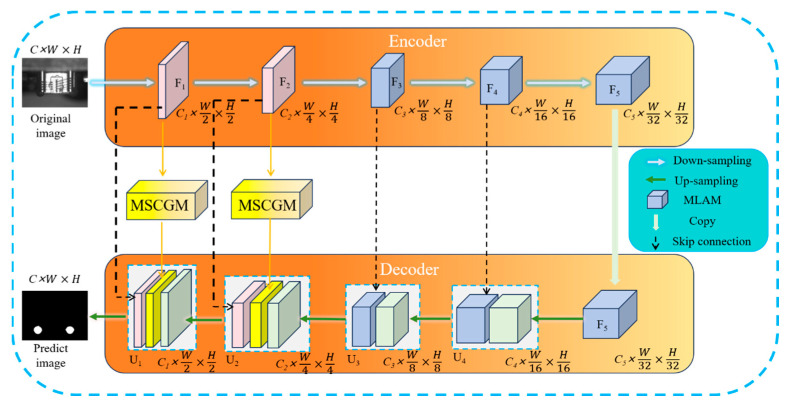
MLSAFNet framework overview. MSCGM: Multi-Scale Channel-Guided Module, MLAM: Multi-Level Attention Module.

Figure 2 presents the model framework of the designed MLSAFNet. It mainly consists of 5 convolutional layers, in which layers 3–5 are embedded with MLAM for enhancing the information extraction ability of the feature maps, and layers 1–2 are connected with MSCGM to ensure that the information of the up-sampling and down-sampling feature maps are fully interacted to increase the target receptive field. MLSAFNet imports important channel features and subtle edge information of adhesive spots into the output feature map by closely linking the encoding stage with the decoding stage. At the same time, based on the U-net network, skip connections are introduced in the up-sampling and down-sampling stages of the network, which greatly enriches the semantic features and location information of the output feature map. For embedded MLAM, the weight enhancement of the features of the adhesive spots region is mainly realized through the fusion of contextual position information at multiple levels. For MSCGM, the information interaction of multi-scale adhesive spots features through channel guidance improves the robustness of recognition and reduces the amount of network parameter computation to improve the network efficiency.

### 2.2. Multi-Scale Channel-Guided Module (MSCGM)

In image recognition, targets have different sizes, shapes, and texture information, and the use of single-scale features may lead to loss of information, whereas multi-scale methods allow the network to focus on a large range of global information at the same time through the fusion of features from different receptive fields. Channel dimensions often contain different high-level semantic information, but not all channels are equally important, and by channel weighting, the network can be guided to pay more attention to key information channels and reduce the interference of redundant information [15,16,17,18]. By fusing multi-scale perception and channel interaction, it is theoretically possible not only to attend to targets at different scales more efficiently, but also to optimize the information flow at the channel level and improve the accuracy of visual detection.

Inspired by the above principle, MSCGM is introduced in MLSAFNet for realizing key information interaction and channel enhancement for multi-scale adhesive spots feature maps to enhance the accuracy and efficiency of adhesive spot segmentation.

**Figure 3:**
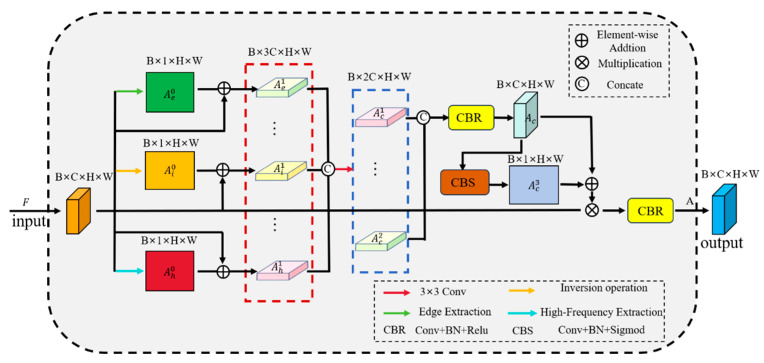
The structure of the Multi-Scale Channel-Guided Module.

As shown in Figure 3, MSCGM is divided into two stages: feature fusion and channel feature interaction. In the feature fusion stage, the background features obtained from inversion, the edge features extracted by the Laplace operator, and the high-frequency features obtained by bilinear interpolation are combined by element addition. This produces a fused feature mapping, which is then passed to the channel feature interaction stage for further processing. The channel interaction part mainly carries out multi-scale channel information interaction, increases the weight value of the channel where the adhesive spot is located, and realizes the separation of the chip background and the foreground of the adhesive spots. Through multi-scale refinement feature fusion and channel interactions, specific extraction of different sizes of adhesive spots features is achieved to improve the detail extraction of feature maps. The detailed steps of MSCGM are as follows:

The input feature map is $F^{\in B,C,H,W}$, first reconstructed based on detail information interaction to obtain ${A_{e}^{0}}^{\in B,1,H,W}$, ${A_{i}^{0}}^{\in B,1,H,W}$*,* and ${A_{h}^{0}}^{\in B,1,H,W}$. Then, the input feature map $F^{\in B,C,H,W}$ is summed over each of the above three feature maps to obtain ${A_{e}^{1}}^{\in B,C,H,W}$, ${A_{i}^{1}}^{\in B,C,H,W}$*,* ${A_{h}^{1}}^{\in B,C,H,W}$ and finally to get $F^{\in B,3C,H,W}$. The specific mathematical calculations are shown below.

(1) $$\left\{\begin{array}{c} A_{e}^{1}=A_{e}^{0}⨁F^{\in B,C,H,W}\\ A_{i}^{1}=A_{i}^{0}⨁F^{\in B,C,H,W}\\ A_{h}^{1}=A_{h}^{0}⨁F^{\in B,C,H,W} \end{array}\right.$$

(2) $$F^{\in B,3C,H,W}=A_{e}^{1}⨂A_{i}^{1}⨂A_{h}^{1}$$

where ⨁ denotes matrix element addition. ⨂ denotes tensor splicing, and $F^{\in B,3C,H,W}$ is the complete detail information interaction feature map that splices $A_{e}^{1}$, $A_{i}^{1},$ and $A_{h}^{1}$. In the channel feature guide stage, $F^{\in B,3C,H,W}$ achieves channel feature reduction to get $F^{\in B,2C,H,W}$ by 3 × 3 convolution to realize the key channel feature weights. This is expressed as in Equations (3)–(5).

(3) $${A_{C}^{1}}^{\in B,C,H,W}=conv3(F^{\in B,3C,H,W})$$

(4) $${A_{C}^{2}}^{\in B,C,H,W}=conv3(F^{\in B,3C,H,W})$$

(5) $$F^{\in B,2C,H,W}=A_{C}^{1}©A_{C}^{2}$$

where conv3 expresses 3 × 3 convolution and © represents tensor splicing. After completing the information feature fusion and channel interaction, in order to further expand the receptive field and enhance the information interaction between feature maps of different scales, further post-processing operations are performed in MSCGM as follows.

(6) $$A_{C}^{\in B,C,H,W}=CBR(F^{\in B,2C,H,W})$$

(7) $${A_{C}^{3}}^{\in B,C,H,W}=CBS(F^{\in B,2C,H,W})$$

(8) $$A={(A}_{C}⨁A_{C}^{3})⨂F$$

where CBR represents 3 × 3 convolution, Batch Normalization, and RELU, and CBS represents 3 × 3 convolution, Batch Normalization, and Sigmoid. The refinement of multi-scale information features and channel interactions are realized using MSCGM, which enhances the weight values of key channels and information in the feature map, and has a positive effect on improving the accuracy of the size segmentation of adhesion spots.

### 2.3. Multi-Level Attention Module (MLAM)

**Figure 4:**
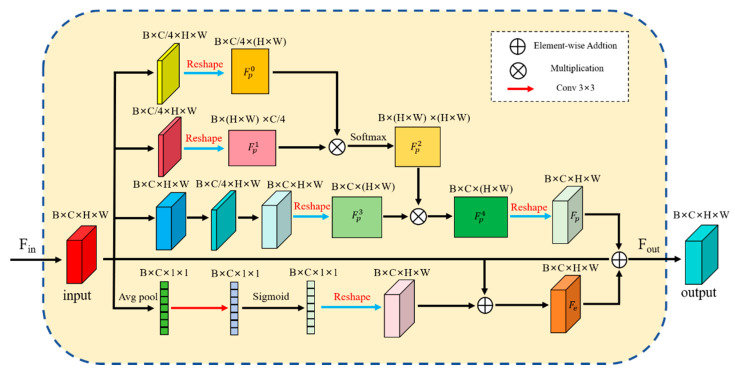
The structure of the Multi-Level Attention Module.

In convolutional neural networks, the predictive performance of the model can be improved by increasing the depth of the network and enhancing the connections between each layer to improve the accuracy of the model [19,20,21]. Although the design method of deepening the number of network layers can improve the accuracy of segmentation, the increase in depth leads to an increase in computational complexity [22]. To reduce the amount of parameter computation and the number of network layers, inspired by human vision attention, Figure 4 designs a Multi-Level Attention Module. MLAMs, on the one hand, improves the computational performance of the network, and on the other hand, it realizes the attention to the location of the adhesive spots, directs the attention to the different layers, and improves the model’s ability to perceive the critical areas. Specifically, we construct a multi-level fusion attention module that extracts and enhances key details through perceptual fusion between key location information of the feature map, enabling the network to efficiently process multi-scale targets. The flow of MLAM is illustrated as follows.

(1) The input feature map $F_{in}$ is transmitted to the upper and lower modules, which are operated in their respective sub-modules, and the computations are Equations (9)–(12).
(2) The results of the operations of the two sub-modules are subjected to matrix addition as shown in Equation (13).

(9) $$F_{P}^{2}=Softmax(Re(conv1(conv3(F_{in})))⨁Re(conv1(conv3(F_{in}))))$$

(10) $$F_{P}^{3}=conv5(conv3(conv1(conv3(F_{in}))))$$

(11) $$F_{p}=Re(F_{P}^{2}⨁F_{P}^{3})$$

(12) $$F_{e}=Re(Sigmoid(conv3(Avgpool(F_{in})))))⨁F_{in}$$

(13) $$F_{out}=F_{e}⨁F_{in}⨁F_{p}$$

where $F_{in}$ represents the input image, $F_{out}$ represents the output image, and where Re represents the feature map reconstruction. conv1, conv3, and conv5 denotes operations with convolutional kernel sizes of 1 × 1, 3 × 3, and 5 × 5, respectively. In the position information fusion section, the input image $F_{in}$ is reconstructed into $F_{P}^{0}$, $F_{P}^{1}$ using a convolution $k_{s}=1$, and for $F_{P}^{3}$, the input $F_{in}$ is processed by convolution kernels of three distinct sizes, namely $k_{s}=1,3,5$. The final output via Equation (11) results in $F_{p}$ fusing multiple positional detail information. For the detail enhancement module, global flat pooling is used first, then 3 × 3 convolution and Sigmoid operation are performed sequentially, and finally it is summed with $F_{in}$ element by element, to get the detail enhancement feature map $F_{e}$. In general, the one-dimensional convolution of the detail enhancement module combined with the elimination of the fully connected layer results in a 33% reduction in computation compared to adding a convolutional and fully connected layer [23]. Therefore, integrating MLAM into the network can reduce the computation of parameters and improve the overall efficiency of the network.

MLAM achieves both extraction and enhancement of semantic information at different levels through a multi-layer attention fusion mechanism, while also reducing redundant computation and improving reasoning efficiency.

### 2.4. Creation of Laser Lens Adhesive Spots (LLAS) Dataset

**Figure 5:**
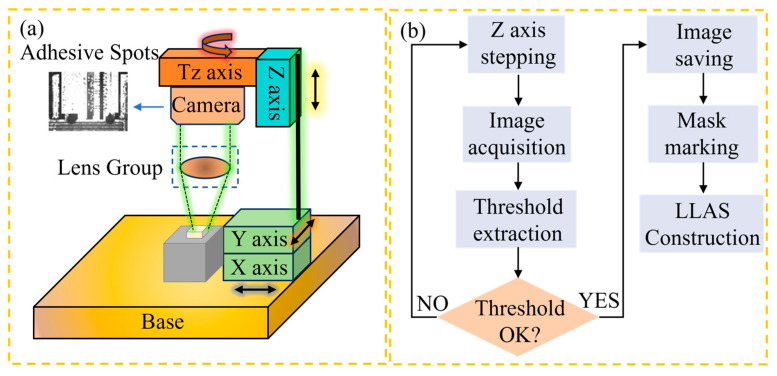
(**a**) Laser chip adhesive spots image acquisition system, (**b**) high-definition adhesive spots image acquisition method.

This paper utilizes 20 dispensing equipment applied in the field for LLAS dataset construction, containing a total of 2800 high-quality images of optical laser chip adhesive spots. Specifically, the Basler 2A2590-60umBAS camera equipped with a microlens package device is used to acquire high-resolution images of the adhesion spots based on a microlens image acquisition algorithm of our own design. A total of 60% of the LLAS dataset is used for training, 20% LLAS for testing, and 20% LLAS for validation. Specifically, the composition of the image acquisition system we designed is shown in Figure 5a.

The adhesive spots area is affected not only by the interference of the laser chip background, but also by the vibration of the optoelectronic packaging equipment, the interference of ambient light, and other uncertain factors that will also have an impact on the clarity of the adhesive spots image. To stably capture the glue spot image and achieve high-precision recognition of the adhesive spot area, the MVL-MY-2-110C-MP telecentric lens is chosen as the telecentric lens module for our dataset image acquisition. As illustrated in Figure 5, the camera is connected to the motion platform of the coupling equipment. The camera is moved to the top of the laser chip via the four-dimensional motion axis. The telecentric lens maintains a constant magnification within the working range of the camera, and its aberration can be disregarded. The relevant parameters of the selected camera and telecentric lens are presented in Table 1.

The clarity of the adhesive spot image directly determines the accuracy of the network’s final segmentation of the adhesive spot; therefore, when performing image acquisition, high-definition images should be acquired as much as possible. For high-power array semiconductor lasers, the chips are arranged in a regular pattern in both the X and Y directions, so the X and Y axes only need to be moved a fixed distance to ensure that the camera is located directly above the laser chip adhesive spots area. At a result, the clarity of the adhesive spots image mainly depends on the accuracy of the Z-axis and the image recognition algorithm. In the process of high-quality, sticky dot image acquisition, we designed an image extraction algorithm based on grayscale thresholding, utilizing the Z-axis 0.1 μm stepping accuracy, and adopting a step-by-step approach to approximate the optimal sharpness threshold to realize the fast acquisition of sticky dot images, and the flow is shown in Figure 5b.

**Figure 6:**
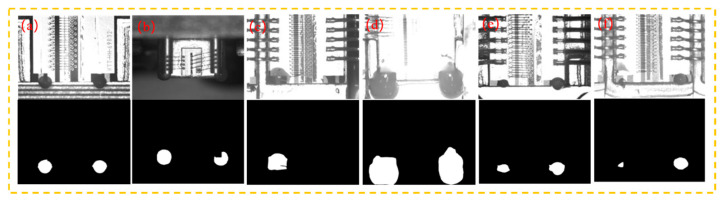
Representative images of each type of adhesive spots in the LLAS.

For the collected image dataset, we mark the area of the adhesive spots pixel-by-pixel, aiming to distinguish the actual dispensing area from the background of the laser chip. Representative images of the glue spots and their labels are given in Figure 6 and Table 2.

Based on the descriptions in Figure 6 and Table 2, it can be seen that the accurate recognition of adhesive spots is mainly affected by the complex background interference and their own multi-scale dimensions. In Seq. (b), it is difficult to distinguish the exact extent of the actual adhesive spots area because the dispensing position required by the laser chip is too close to the gray value of the image of the adhesive spots. In Seq. (c), the recognition of adhesive spots is mainly affected by the highlighted background, making it difficult to distinguish the background from the target. There is a huge gap in the scale of the adhesive spots in Seq. (e) and (f), resulting in tiny focuses that are susceptible to background interference and missed detection.

As a result, when it comes to detecting the glue spot area after laser coupling and dispensing are finished, given the diverse shapes of the spots area and complex background interference, a special network design is necessary to achieve accurate recognition. The LLAS dataset employs a binary classification approach to segment the target exclusively from the background of the laser chip, while integrating a deep learning model to enhance the efficiency of adhesive spots area segmentation.

## 3. Experimental and Results

In this section, we primarily demonstrate the reliability of MLSAFNet through ablation experiments of each module and comparison with existing advanced networks. Firstly, we determine the loss function and optimization function for model training, as well as the performance indicators for evaluation and comparison. Secondly, in accordance with the network structure of MLSAFNet, ablation experiments are conducted on the added modules in stages to verify their effectiveness. Thirdly, we compare MLSAFNet with other advanced network models to highlight the advantages of our proposed network in terms of the output effect and performance indicators of segmentation. Finally, the parameters of the adhesive spots detected by MLSAFNet are extracted and compared with the mask to test the feasibility of its final application.

### 3.1. Experimental Conditions

The experiment is performed on a computer outfitted with a 12 GB NVIDIA RTX 3060 GPU and is executed with the assistance of the PyTorch 1.10.2 framework. Adam is chosen as the regularization optimizer, and the learning rate is set at 10^−4^. Additionally, to increase the number of adhesive spots images, the dataset is processed by image enhancement technology during training. To expedite the convergence speed of the network, we combine the binary cross-entropy (*BCE*) loss and the Dice loss function to construct a new loss function, as follows.

(14) $$BCE=-\frac{1}{N}\sum_{i=1}^{N} [y_{i}\log({\hat{y}}_{i})+(1-y_{i})\log(1-y_{i})]$$

(15) $$\bar{Dice}=1-\frac{2\sum \left|A\cap B\right|}{\sum \left|A|+|B\right|}$$

(16) $$Loss=\alpha \cdot BCE+(1-\alpha )\cdot \bar{Dice}$$

In this context, α is a hyperparameter used to balance the contributions of the two loss functions, set to 0.5. $y_{i}$ denotes the ground truth labels and ${\hat{y}}_{i}$ denotes the predicted values, while A and B correspond to the predicted and actual foreground regions, respectively.

To quantitatively assess the performance of the proposed network, commonly used metrics in image recognition were utilized, including the *F*_1_ score, mean intersection over union (mIoU), and Dice coefficient [24,25,26,27]. The Dice coefficient quantifies the similarity between the predicted outcomes and the ground truth mask, where values approaching 1 reflect enhanced network performance. The detailed methodology for the numerical evaluation is presented as follows.

(17) $$F_{1}=\frac{2P_{r}\times R_{e}}{P_{r}+R_{e}}$$

(18) $$P_{r}=\frac{TP}{TP+FP}$$

(19) $$R_{e}=\frac{TP}{TP+FN}$$

(20) $$\mathrm{mIoU}=\frac{1}{n}\sum_{i=0}^{n}\frac{TP}{FN+FP+TP}$$

(21) $$\mathrm{Dice}=\frac{2\cdot TP}{2\cdot TP+FP+FN}$$

In the equation, True positives (*TP*) denotes the number of samples correctly classified as positive, False positives (*FP*) represents the number of samples incorrectly classified as positive, and False negatives (*FN*) refers to the number of samples incorrectly classified as negative. Whatmore, the receiver operator characterization (ROC) curve can reflect the relationship between the True positive rate (TPR) and the False alarm rate (FAR), and the larger the area enclosed by its curve proves that the network performance is more powerful, and we use the ROC curve to visually and numerically evaluate different networks [28].

(22) $$\mathrm{TPR}=\frac{TP}{TP+FN}$$

(23) $$\mathrm{FAR}=\frac{FP}{FP+TN}$$

### 3.2. Ablation Experiments

**Figure 7:**
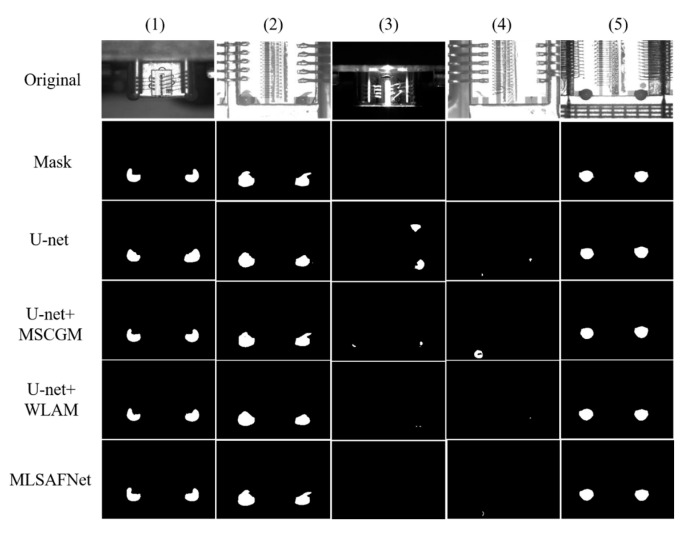
Comparison of the visualization results of the ablation experiments for MLASFNet.

Ablation studies clearly demonstrate the contribution of each module in the network. In this study, we integrated each module into the U-net framework in turn and then compared the results through numerical analysis and visual evaluation. The experimental results are shown in Table 3 and Figure 7.

Analysis of Table 3 reveals that the numerical evaluation metrics for the U-net alone achieved *F*_1_, *mIoU*, and *Dice* scores of 87.31%, 87.24%, and 93.10%, respectively. This may be due to the high number of high-resolution images in LLAS, resulting in segmentation results that still achieve high-quality accuracy when only U-net is used. The introduction of the MSCGM module enhances performance metrics, improving the *F*_1_, *nIoU*, and *Dice* scores by 0.69%, 1.05%, and 0.60%, respectively. Although MSCGM has limited improvement on the overall performance of the network, it can still be seen from the numerical evaluation results that MSCGM enhances the feature refinement and channel interactions, which improves the accuracy of adhesive spot segmentation to some extent. When the U-net + SCFM network structure is used, compared with U-net, the *F*_1_, *mIoU*, and *Dice* are improved by 1.25%, 2.5%, and 1.43%, respectively. Compared to U-net + MSCGM, U-net + MLAM demonstrates an obvious performance improvement. Specifically, the U-net + MLAM, which incorporates a muti-level attention module, achieved increases of 0.56%, 1.45%, and 0.83% in the *F*_1_ score, *mIoU*, and *Dice* coefficient, respectively. While using MSCGM and MLAM individually provides limited improvements to the overall network performance, integrating both modules into MLSAFNet led to increases of 1.84%, 3.91%, and 2.21% in *F*_1_ score, *mIoU*, and *Dice* coefficient, respectively. Although MLSAFNet does not have a significant improvement in effect relative to the baseline network, the small improvement effect for microscale adhesive spots will also demonstrate significant optimization results in industrial applications.

Figure 7 illustrates the individual effects of each module on the performance of adhesive spot segmentation from a visual display perspective. For Seq. (1) and (2), when the adhesive spots are segmented using only U-net, the segmentation accuracy is low, although the adhesive spots region can be localized correctly. When U-net + MSCGM is used, the shape of the glue spot can be segmented more accurately by multi-scale feature refinement, but there is still a low false detection rate. When U-net + MLAM is used, although the attention mechanism makes the segmentation focus on the adhesive spots region, its edge detail delineation is not obvious, resulting in a certain false detection rate in the results. When MSLAFNet is further used, the fusion of multi-scale feature interaction and multi-level attention mechanism results in high accuracy of the adhesive spot shape detection results. For Seq. (3) and (4) with prominent background interference and without adhesive spots on chips, the U-net has a high false detection rate and produces false alarms in the absence of adhesive spots, which may be due to the lack of an attention mechanism, resulting in the inability to focus on the critical region. U-net + MSCGM, due to the use of feature refinement and channel interaction only, still shows a higher false alarm rate, although the positive detection rate is somewhat improved compared to U-net. The attention mechanism is employed in U-net + MLAM, which effectively reduces the weight of irrelevant regions and exhibits only a low false alarm rate. MSLAFNet, on the other hand, combines the advantages of MSCGM and MLAM to further minimize the false alarm rate and improve the high correctness of the detection results. In Seq. (5), each network combination shows high-quality adhesive spot segmentation results, which may be caused by the high image clarity of Seq. (5) with a clear contrast between target and background.

The above numerical and vision evaluation results proved that by utilizing the multi-scale channel guidance and multi-level attention mechanism, as the network focus can be concentrated on the key channels and target pixel regions, which effectively improves the weight values of the key channels and focus regions, and significantly enhances the segmentation ability of the neural network on the adhesive spot region.

**Figure 8:**
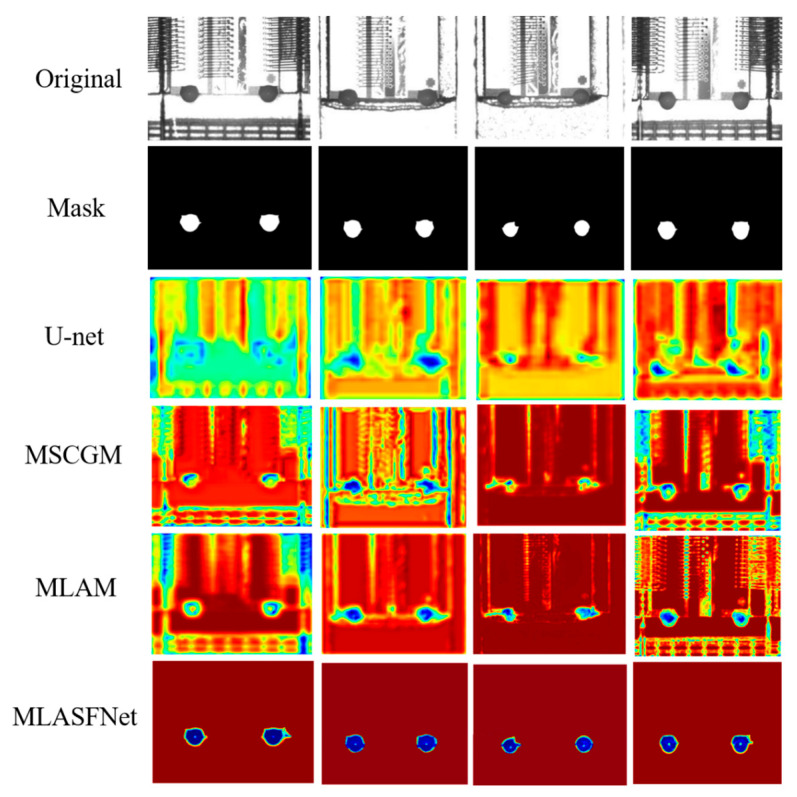
Comparison results of visualized hotspot maps with the addition of different modules.

The use of hotspot maps allows for a more intuitive display of the role of each module in MSLAFNet and enhances the interpretability of the network. Figure 8 shows the evolution of the hotspot feature map based on each module to reflect the specific effects of MSCGM and MLAMs in the network. When only U-net is used, the detection results are concentrated in a wide range of areas centered on the adhesive spot and are heavily interfered by the background. MSCGM eliminates most of the background interference by refining the information and assigning weight values to the key channels. MLAM, on the other hand, utilizes the attention mechanism to accurately locate the adhesive spot area, and ultimately achieves the precise segmentation of the adhesive spots area.

### 3.3. Comparison Experiments

Currently, deep learning networks based on adhesive spots detection have not been widely reported, and we use the current state-of-the-art infrared small-target segmentation algorithm to compare with MLSAFNet to comprehensively evaluate the performance of the network. UIU embeds a small U-net into the U-net backbone network, enabling multi-level and multi-scale characterization learning of targets [29]. LSPM utilizes a powerful pyramid structure and attentional mechanisms to segment targets [30]. DNA enables target segmentation by combining densely nested interaction modules and channeled spatial attention modules [31]. The MTU network constructs a multi-level feature extraction module based on a vision transformer (ViT) convolutional neural network, which realizes the detection of long-range targets [32]. MRF^3^Net combines multiple receptive fields with an effective feature fusion strategy for efficient infrared small-target detection [33]. The evaluation metrics of each network are compared in Table 4.

The results of the numerical comparison of the six algorithms in Table 4 show that MLSAFNet has more obvious advantages. Compared to the top-performing metrics of other networks, MLSAFNet achieved improvements of 0.99% in *F*_1_, 1.07% in *mIoU*, and 0.58% in *Dice*. MTU’s segmentation is second only to MLSAFNet, due to the fact that it embeds a convolutional neural network with a visual transformer and a multi-level feature extraction module, which allows for feature refinement and concentration of regional attention. However, UIU uses simple U-net network nesting, which is unable to accomplish multi-scale adhesive spot region focusing, thus leading to its poor numerical evaluation results. The pyramid mechanism of the LSPM is poor performance for adhesive spot segmentation containing multiple targets and only outperforms the UIU. MRF^3^Net and DNA used a multi-scale interaction strategy, which performed moderately well on the results of adhesive spot segmentation. In the comparison of image segmentation speed, which is more concerned by automation equipment, the processing speed of MLSAFNet is only 3.55 s per 100 images, which is better than the processing speed of other algorithms. Improving segmentation speed significantly boosts the efficiency of recognizing adhesive spots areas, which is crucial for accelerating automated production of lasers. Compared to MLSAFNet, MTU is 0.17 s slower, while LSPM has the slowest processing time at 64.06 s.

**Figure 9:**
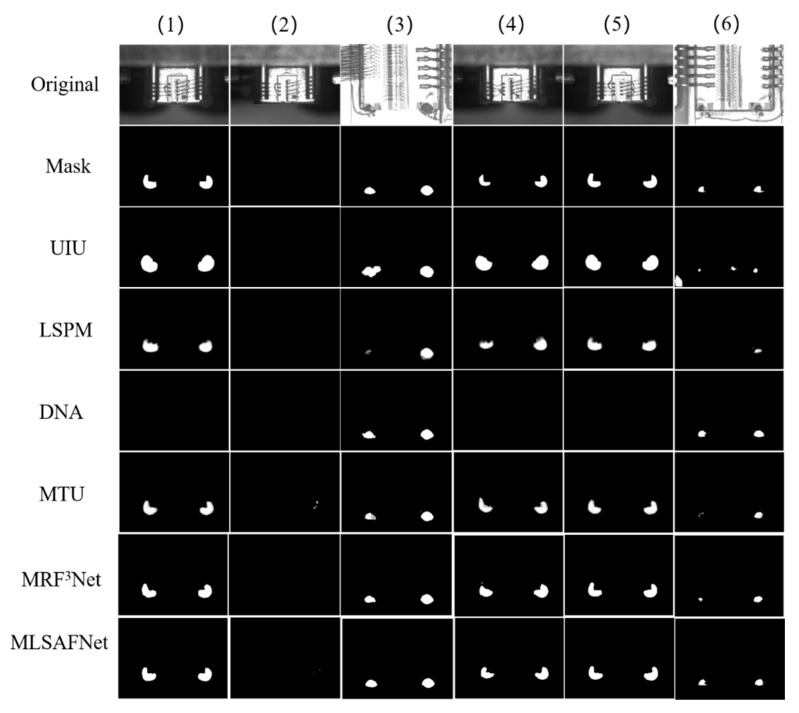
Comparison of detection results of six networks.

Figure 9 visualizes the comparison of the output results of the six networks. Seq. (1)–(6) place semiconductor laser chips of different models and different adhesive spots situations. In Seq. (3) and (6), when faced with complex backgrounds and varying sizes of adhesive spots areas, the two networks UIU and LSPM fail to accurately segment the adhesive spot regions. The visualization results of the above even images show that in the face of target segmentation under complex background, simple network nesting or a small amount of information interaction is less robust and cannot accurately detect the target under strong background interference. When confronted with Seq. (1) and (5), the DNA showed severe leakage, which may be due to too dark and weak adhesive spots affecting the network segmentation performance of the DNA. In Seq. (2), there is a false alarm in the MTU, which may be due to the fusion of too many information details leading to an overfitting phenomenon that affects the accuracy of the detection. The MRF^3^Net multisensory field combined with an effective feature fusion strategy can be effectively localized to the adhesive spots region, but still lacks accuracy for detailed segmentation of adhesion points, such as Seq. (1) and (4). Compared to the other five networks, the MLSAFNet, which is fused on the MSCGM and MLAM, demonstrates greater precision in segmenting the contours of the adhesive spot area, which is important for the recognition of the edge contour of the adhesive spots region in the subsequent preprocessing.

**Figure 10:**
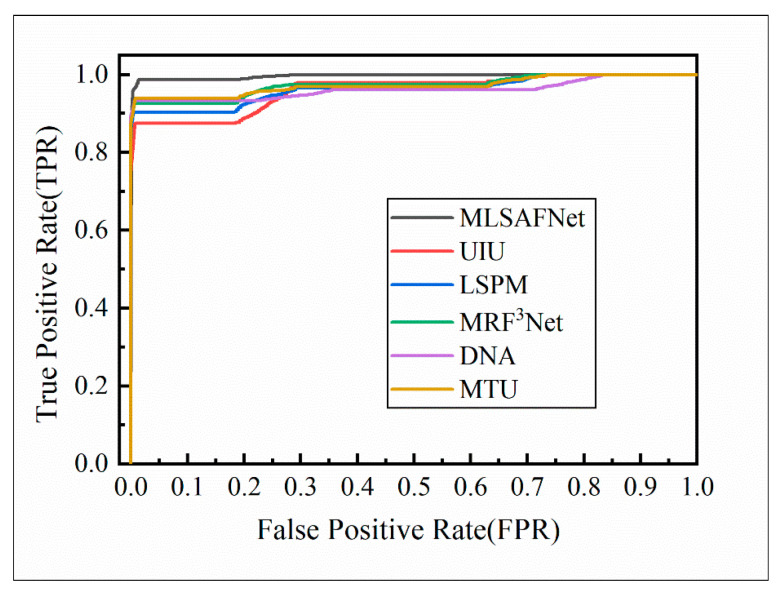
ROC curves for six methods based on LLAS dataset.

The ROC curve offers a clear method for analyzing the trade-offs between True positive rates and False positive rates at various thresholds, allowing for a comprehensive understanding of the neural network’s performance by examining the shape of the curve and the area under the curve (AUC). The ROC curves of the six comparative models on the glue spot dataset are illustrated in Figure 10. From the results in Figure 10, it can be seen that MLSAFNet has the largest enclosing area, which indicates that the multi-scale channel guidance and multi-level attention fusion mechanism help to accurately segment the adhesive spots and improve the performance of the network.

### 3.4. Adhesive Spots Feature Extraction Experiment

**Figure 11:**
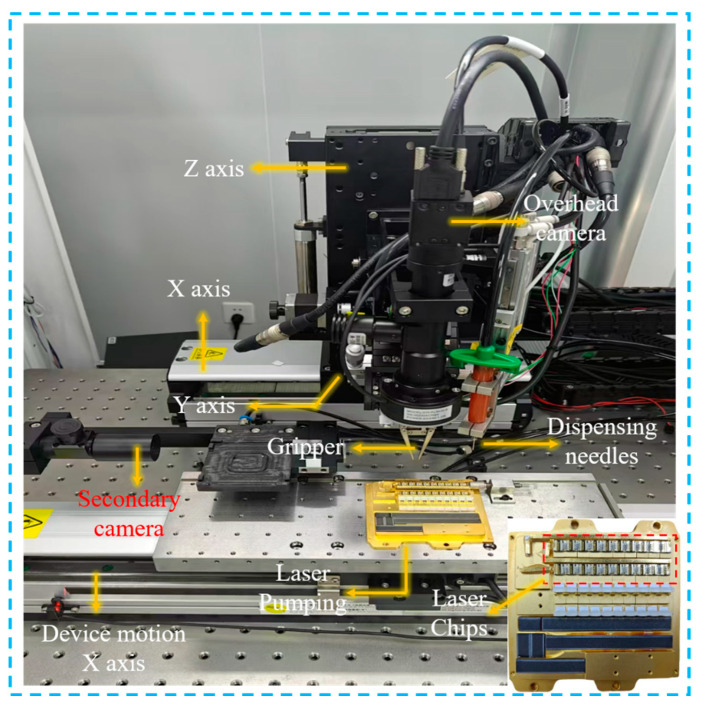
Dispensing and adhesive spots detection system for microlens packaging.

By improving the detection accuracy of the adhesive spots and providing early warning for the package results of the microlens, the beam quality of the high-power laser can be effectively improved. Figure 11 illustrates automated equipment for microlens packaging, including a precision dispensing system and an adhesive spot detection system.

The system is mainly used to realize the coupling and package of collimated microlenses, through the use of qualified adhesive spots for UV curing of collimated microlenses, to achieve the purpose of high-quality output of the laser beam. Specifically, first, a high-resolution image of the adhesive spots in the chip area is extracted by an overhead camera, and the adhesive spots are detected and parameters are extracted using MLSAFNet, and then, after the adhesive spot is detected and qualified, the collimated microlens is clamped with a gripper for optical coupling and UV curing, so as to complete the high-quality output of the collimated beam.

**Figure 12:**
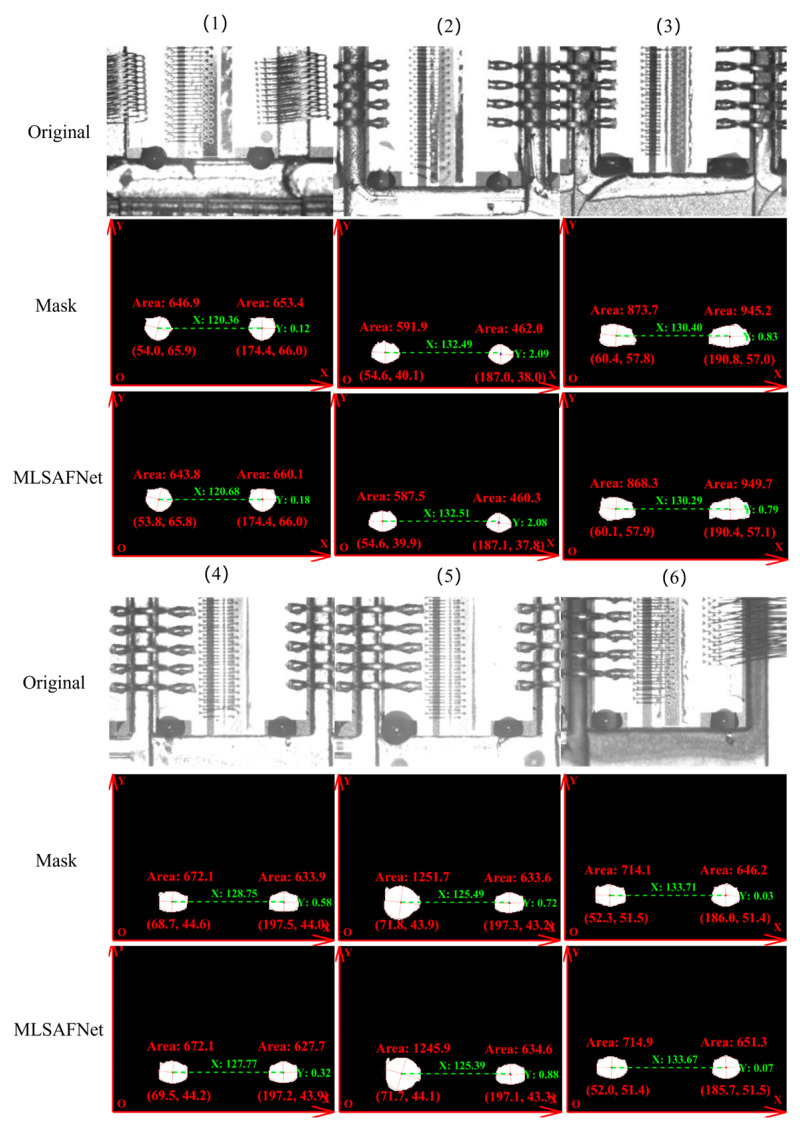
Comparison of detection accuracy of adhesive spots area and position.

Hough ellipse fitting is commonly utilized for various detection tasks [33,34,35]. In Figure 12, the Hough ellipse fitting algorithm is used to identify the elliptical contours of the edges of the region of the adhesive spots as well as the center coordinates. Seq. (1)–(6) show pictures of multi-scale glue dots under different backgrounds and types of chips, which are interfered by different intensities of light, respectively, leading to visual difficulties in distinguishing features for the human vision. The comparison with the glue spot label images indicates that the maximum errors in the recognition of the position and area of the adhesive spots region by MLSAFNet after segmentation are 21.83 μm and 0.003 mm^2^, respectively. The experimental results show that MLSAFNet combining MSCGM and MLAM can effectively achieve accurate segmentation and localization of the adhesive spots region. Finally, based on the segmentation results of MLSAFNet on the adhesive spot, the quality of the adhesive spot is judged and the glue replenishment operation is performed. After hundreds of experiments, the results show that the success rate of microlens cementation is more than 96%.

## 4. Conclusions

The novelty of our research is mainly reflected in the fact that an industrial dataset of multi-scale micro adhesive spots is constructed for the first time based on the self-developed collimated mirror package equipment, and we propose adopting a deep learning strategy to realize the high-precision detection of micro adhesive spots, which achieves a detection accuracy of 91.15%. In the subsequent microlens packaging, the success rate of microlens package guided by MLSAFNet detection results reached 96%. For labor, our method provides a new idea for the precise assembly of microscale optical components in optoelectronic devices.

However, since our laser chip adhesive spot dataset is only for dual adhesive spot images in pump source lasers, it leads to a lack of diversity in the spotting situations and a high recognition success rate, which is a great limitation for image recognition in multiple types of adhesive spots situations. In our future research, we will collect more images of adhesive spots with different dispensing situations and backgrounds of different types of pump sources and lasers chips to build up a larger-capacity dataset of dispensing situations for the field of automated inspection. At the same time, we will study lighter and more efficient algorithms for adhesive spots area recognition to achieve fast and accurate recognition of adhesive spots areas in the automated coupling of laser devices.

## Figures and Tables

**Table 1:** Parameters of industrial cameras and telecentric lens.

Industrial Cameras		a2A2590-60umBAS	
Resolution	2592 × 1944	Frame rate	60 fps
Pixel size	2 μm × 2 μm	Signal-to-noise ratio	38.7 dB
Telecentric lens MVL-MY-2-110C-MP			
Working distance	110 mm	Magnifying power	2.0
Image size	Φ11 mm	Telecentricity	0.1°

**Table 2:** Characteristics of laser chip adhesive spots.

Seq.	Image Size (Pixels)	Adhesive Spot Area Characterization
a	256 × 192	Strong background Standard adhesive spots
b		Complex background Weak target
c		Soothing background Irregular single target
d		High-light background Irregular huge target
e		Complex background Irregular and tiny target
f		Soothing background Tiny target

**Table 3:** MLSAFNet ablation experiments.

U-net	MSCGM	MLAM	mIoU (%)	Dice (%)	*F*_1_ (%)
√	×	×	87.24	93.10	87.31
√	√	×	88.29	93.70	88.00
√	×	√	89.74	94.53	88.56
√	√	√	91.15	95.31	89.15

**Table 4:** Comparison of six state-of-the-art methods for adhesive spots detection.

Method	mIoU (%)	Dice (%)	*F*_1_(%)	Time (s/100 Images)
UIU	75.94	86.21	78.92	7.92
LSPM	77.74	87.34	83.00	64.06
DNA	84.33	91.30	85.48	11.13
MTU	90.08	94.73	88.16	3.72
MRF^3^Net	85.33	84.41	83.78	9.61
MLSAFNet	91.15	95.31	89.15	3.55

## Data Availability

The datasets used and analyzed during the current study are available from the corresponding author on reasonable request.
